# Intake of a Ketone Ester Drink during Recovery from Exercise Promotes mTORC1 Signaling but Not Glycogen Resynthesis in Human Muscle

**DOI:** 10.3389/fphys.2017.00310

**Published:** 2017-05-23

**Authors:** Tijs Vandoorne, Stefan De Smet, Monique Ramaekers, Ruud Van Thienen, Katrien De Bock, Kieran Clarke, Peter Hespel

**Affiliations:** ^1^Exercise Physiology Research Group, Department of Kinesiology, KU LeuvenLeuven, Belgium; ^2^Laboratory of Exercise and Health, Department of Health Sciences and Technology, ETH ZurichZurich, Switzerland; ^3^Department of Physiology, Anatomy and Genetics, University of OxfordOxford, United Kingdom

**Keywords:** ketone, exercise recovery, muscle, human, C2C12 myotubes, protein, glycogen, mTORC1 signaling

## Abstract

**Purpose:** Ketone bodies are energy substrates produced by the liver during prolonged fasting or low-carbohydrate diet. The ingestion of a ketone ester (KE) rapidly increases blood ketone levels independent of nutritional status. KE has recently been shown to improve exercise performance, but whether it can also promote post-exercise muscle protein or glycogen synthesis is unknown.

**Methods:** Eight healthy trained males participated in a randomized double-blind placebo-controlled crossover study. In each session, subjects undertook a bout of intense one-leg glycogen-depleting exercise followed by a 5-h recovery period during which they ingested a protein/carbohydrate mixture. Additionally, subjects ingested a ketone ester (KE) or an isocaloric placebo (PL).

**Results:** KE intake did not affect muscle glycogen resynthesis, but more rapidly lowered post-exercise AMPK phosphorylation and resulted in higher mTORC1 activation, as evidenced by the higher phosphorylation of its main downstream targets S6K1 and 4E-BP1. As enhanced mTORC1 activation following KE suggests higher protein synthesis rates, we used myogenic C_2_C_12_ cells to further confirm that ketone bodies increase both leucine-mediated mTORC1 activation and protein synthesis in muscle cells.

**Conclusion:** Our results indicate that adding KE to a standard post-exercise recovery beverage enhances the post-exercise activation of mTORC1 but does not affect muscle glycogen resynthesis in young healthy volunteers. *In vitro*, we confirmed that ketone bodies potentiate the increase in mTORC1 activation and protein synthesis in leucine-stimulated myotubes. Whether, chronic oral KE intake during recovery from exercise can facilitate training-induced muscular adaptation and remodeling need to be further investigated.

## Introduction

Skeletal muscle tissue plays a pivotal role in the regulation of whole body metabolism, functional work capacity, and exercise performance (Zurlo et al., 1994; Egan and Zierath, 2013). While no medicine has proven to be more efficient than exercise to improve muscle function (Booth et al., 2000), exercise-nutrition interactions significantly impact on energy substrate replenishment and on muscle remodeling and repair post-exercise (Beelen et al., 2010; Hawley et al., 2011). During recovery from exercise, amino acid intake is required to stimulate muscle protein synthesis (Biolo et al., 1997; Churchward-Venne et al., 2012), whilst high-rate carbohydrate intake promotes resynthesis of glycogen stores (Costill et al., 1981; Burke et al., 2016). Furthermore, improving the (nutritional) conditions under which exercise promotes muscle protein synthesis is not only important for athletes, but also in clinical conditions requiring enhanced anabolic response in patients who are unable to perform high-intensity exercise.

Skeletal muscle protein synthesis following exercise is mainly regulated by the mechanistic target of rapamycin complex 1 (mTORC1) (Bodine et al., 2001; Schiaffino and Mammucari, 2011). mTORC1 regulates protein translation initiation and elongation via phosphorylation of the ribosomal protein S6 kinase 1 (S6K1) and the eukaryotic initiation factor 4E-binding protein 1 (4E-BP1) (Burnett et al., 1998). Muscle contractions *per se* activate mTORC1 in a stress and/or mechanic-related manner, but also the intake of proteins activates mTORC1 and protein synthesis (Morton et al., 2015). On the other hand, as protein synthesis is a high ATP-consuming process, mTORC1 activity is inhibited during periods of energetic stress by the AMP-activated protein kinase (AMPK; Bolster et al., 2002; Dreyer et al., 2006). AMPK is the principal energy sensor in muscle cells and is activated by decreased energy levels during and after contractions (Hardie, 2011). AMPK promotes energy substrate catabolism to generate ATP, and at the same time inhibits anabolic processes requiring ATP, in order to preserve energy status (Gwinn et al., 2008). During and after strenuous exercise, activation of AMPK is responsible for the delayed activation of muscle protein synthesis (Dreyer et al., 2006; Thomson et al., 2008). Rapid restoration of muscle energy balance to de-activate AMPK is important in that it enhances post-exercise mTORC1 activation, protein synthesis and eventually training-induced muscle remodeling.

Muscle glycogen is the primary fuel for muscle contractions during high-intensity endurance exercise. Glycogen stores are small relative to the total energy need in prolonged exercise, which makes initial muscle glycogen content an important determinant of performance (Hawley et al., 1997). Hence, maximizing muscle glycogen resynthesis is crucial for maintenance of endurance exercise performance whenever time for recovery between events is short relative to the time needed to full glycogen repletion (Burke et al., 2016).

Acetoacetate (AcAc) and beta-hydroxybutyrate (βHB) are the main ketone bodies (KB) produced in the liver in response to low blood glucose and insulin levels, and play a critical role in survival during episodes of fasting. Three weeks of starvation result in blood ketone levels of up to ~6 mM (Cahill, 2006). Alternatively, a strict ketogenic diet containing low carbohydrate, low protein, but high fat (Cahill and Veech, 2003), can also elevate circulating KB levels to ~1–2 mM within 2–4 days (Pinckaers et al., 2016). Under those conditions, KB serve as circulating energy substrates for metabolic tissues such as the brain (Kashiwaya et al., 2010), cardiac (Nakatani et al., 2015), and skeletal muscle (Cahill, 2006). Mechanistically, KB fuel the tricarboxylic acid (TCA) cycle after being converted to acetyl-coenzyme A (Shi and Tu, 2015; Murray et al., 2016), and prevent the depletion of carbohydrate stores as well as the breakdown of muscle contractile proteins for gluconeogenesis and TCA cycle anaplerosis. While KB in this way could potentially affect performance, the dietary conditions needed to stimulate endogenous KB synthesis are not compatible with optimal performance in most sports (Cox and Clarke, 2014). Recently, the ketone monoester (R)-3-hydroxybutyl (R)-3-hydroxybutyrate (hereafter referred to as KE) has emerged as an alternative approach to raise blood KB levels in the absence of any other dietary modification. Ingestion of KE increases blood ketone appearance within minutes of consumption (Clarke et al., 2012b), leading to circulating concentrations similar to those observed in prolonged fasting, even in the presence of elevated blood insulin levels (Clarke et al., 2012b; Cox et al., 2016). KE enhanced endurance exercise performance in athletes, at least partly because KB act as an alternative substrate for oxidative phosphorylation (Cox et al., 2016). Interestingly, by reducing the need for glucose-derived ATP production, KE also resulted in glycogen sparing during exercise (Cox et al., 2016).

Against the above background, we decided to investigate whether nutritional ketosis, induced via KE ingestion, could enhance markers of muscle protein synthesis and/or glycogen resynthesis during recovery from strenuous exercise. While ingestion of KE did not increase muscle glycogen resynthesis, it increased activation of muscle protein synthesis markers. Using an *in vitro* strategy, we confirmed that KB potentiated leucine-mediated mTORC1 activation and that this resulted into higher rate of leucine-mediated protein synthesis. Altogether, our data show that KE enhances mTORC1 activation during recovery from exercise but does not affect muscle glycogen resynthesis in human muscle.

## Methods

This work involved both a study in healthy human volunteers and a series of *in vitro* experiments in skeletal muscle myotubes.

### Study in healthy volunteers

#### Subjects

Eight healthy, young male volunteers [height: 181.9 ± 1.8 cm; body weight (BW): 76.6 ± 2.7 kg; body mass index: 22.9 ± 0.7 kg/m^2^; age: 20–24 years old; one repetition maximum (1RM): 75 ± 1.25 kg] participated in the study, which was approved by the KU Leuven Biomedical Ethics Committee (B322201316517), and conformed to the Declaration of Helsinki. All subjects were involved in regular physical activity, but none participated in elite competitive sports. Potential participants were screened using a health questionnaire and underwent a medical examination, including a resting ECG, prior to participation in this study. Subjects were instructed not to change their normal diet or physical activity level during the study, and gave their written consent after having been informed of all experimental procedures and risks associated with the experiments. During the study, one subject dropped-out due to a knee-injury that was unrelated to the study protocol. The subject was excluded from all analysis.

#### Study design (Figure 1)

The study was of a randomized, double blind, placebo-controlled crossover study design involving two experimental sessions with a 3-week washout period in between. Each experimental session consisted of a glycogen-depleting exercise protocol with the right leg, followed by a 5 h recovery period during which a high-dose protein-carbohydrate mixture was administered. In addition, in one experimental condition the subjects received a KE drink during the recovery period, whilst in the other condition they received an isocaloric PL drink (Clarke et al., 2012b). Two weeks before the start of the study, the subjects participated in two preliminary sessions with a 1-week interval in between to become familiar with the experimental procedures and to adjust the individual exercise workloads to be used in the experimental sessions. These sessions were also used to determine the 1-repetition-maximum (1RM) for knee-extension exercise.

**Figure 1:**
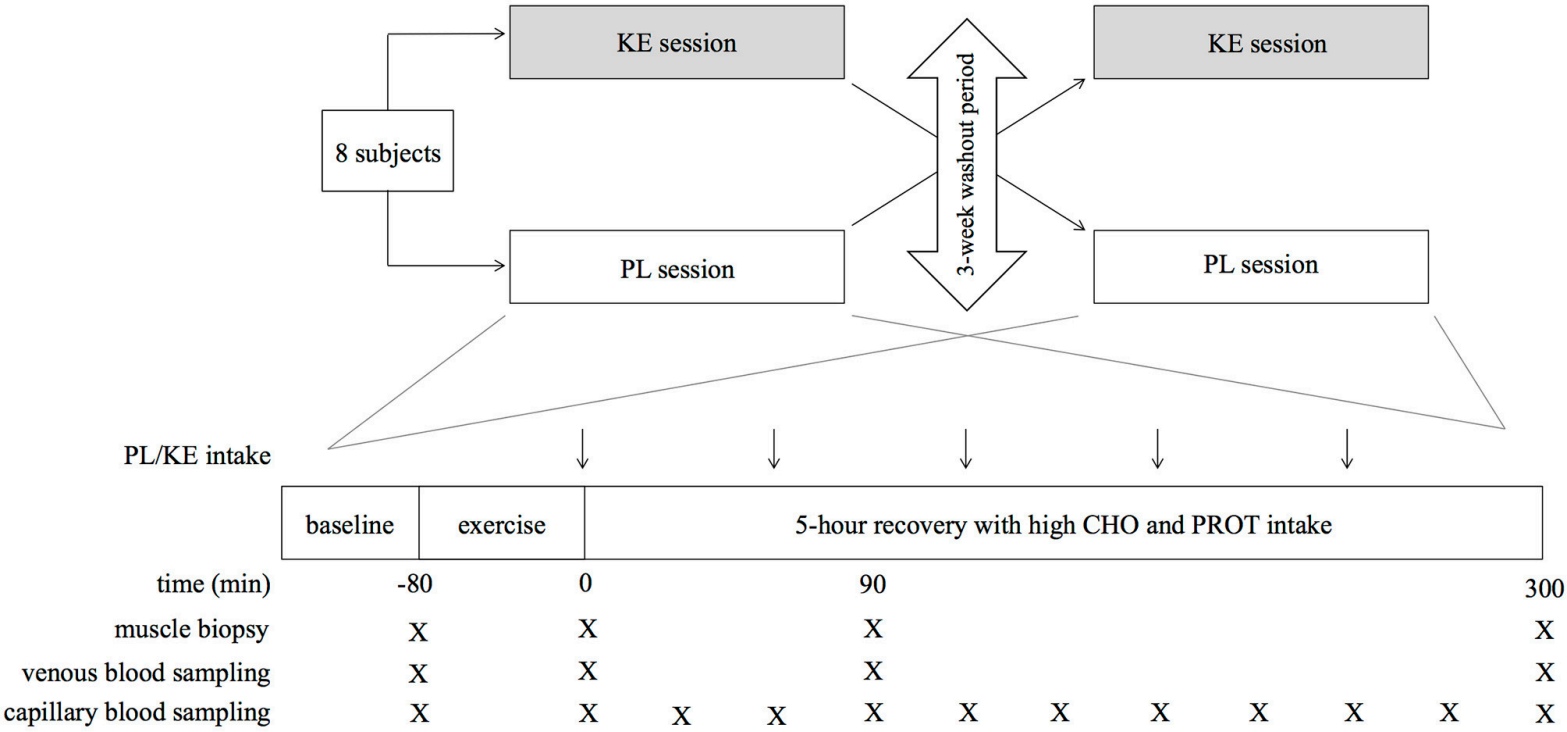
**Schematic representation of the study design**. PL, placebo; KE, ketone ester; KE, ketone ester (R)-3-hydroxybutyl (R)-3-hydroxybutyrate; CHO, carbohydrate; PROT, protein. Arrows (↓) indicate ingestion of PL or KE. Negative time points indicate before exercise; positive time points indicate after exercise.

#### Experimental sessions

The evening before each experimental session the subjects reported to the laboratory for a standardized dinner (18 kcal·kg^−1^ BW, 70% carbohydrates, 15% protein, 15% fat). Next morning, they received a standardized breakfast (10 kcal·kg^−1^ BW, 70% carbohydrates, 18% protein, 12% fat) and, later lunch (10 kcal·kg^−1^ BW, 70% carbohydrates, 18% protein, 12% fat). After lunch the subjects rested for 2 h in a comfortable chair before blood samples were taken from both an earlobe (capillary blood) and an arm vein (Venoject, Tokyo, Japan). A percutaneous needle biopsy for baseline determinations was then taken from m. vastus lateralis in the left leg as described below. Post-exercise biopsies later in the experiment were taken from the other leg, to limit artifacts due to multiple biopsies in the same muscle belly (Van Thienen et al., 2014). Thereafter, warming-up was started by unilateral cycling (50-70-90 Watt, 4 min each) with the right leg on a cycle ergometer (Avantronic Cyclus II, Leipzig, Germany), followed by 2 series of 30 unilateral knee-extensions at 10% of 1RM on a self-constructed isokinetic dynamometer (Hespel et al., 2001). The subjects then started an intermittent exercise protocol aimed to deplete muscle glycogen in the right leg by unilateral knee-extensions (70°–130° knee-angle) at a rate of 0.5 Hz. Subjects first performed an exercise bout, during which they produced as high as possible a mean power output for 5 min. Thereafter they did 9 series of 30 knee-extensions at 30% of 1RM, followed by 5 series of 6 contractions at 70% of 1RM. The contraction series were interspersed by 30 s passive rest intervals. Immediately after the last exercise a capillary blood sample was taken from the earlobe, and a percutaneous needle biopsy was taken from m. vastus lateralis. Following this post-exercise biopsy, the subjects actively recovered by unilateral cycling for 20 min at 50 Watt (Avantronic Cyclus II, Leipzig, Germany), after which they rested in a comfortable chair in the laboratory till 5 h post-exercise. After 90 min and 5 h of recovery another needle biopsy was taken from m. vastus lateralis. During recovery capillary blood samples from the earlobe were taken at 30-min intervals, and a venous sample was collected from an arm vein at 90 min and 5 h. At the end of each experimental session, the subjects filled out a questionnaire to assess gastro-intestinal tolerance (adapted from Pfeiffer et al., 2009). The questionnaire was organized in three sections. Upper abdominal problems (reflux, bloating, nausea, vomiting); lower abdominal problems (cramps, flatulence, abdominal pain, diarrhea); and systemic problems (dizziness, headache, muscle cramp, urge to urinate).

#### Nutritional supplementation during recovery

In order to elicit optimal rates of muscle protein and glycogen synthesis after exercise, subjects received a high-dose protein–carbohydrate drink throughout the recovery period (Table 1). These drinks (250 ml aliquots) were ingested at 30-min intervals starting immediately after exercise and delivered 1 g·kg^−1^ BW·h^−1^ carbohydrates (65% maltodextrin, 35% dextrose) plus 0.3 g·kg^−1^ BW·h^−1^ hydrolyzed whey-protein concentrate (Body&Fit, Heerenveen, The Netherlands) yielding a total leucine intake of 12.1 ± 0.4 g per person during the 5 h recovery period. In one session, the subjects also drank a KE supplement [>96% (R)-3-hydroxybutyl (R)-3-hydroxybutyrate] to elevate circulating plasma ketone concentrations (Clarke et al., 2012a,b; Cox et al., 2016). In the other session, they received an isocaloric PL supplement containing >96% long chain triglycerides and which was similar to KE in taste and appearance, as described previously (Cox et al., 2016). Immediately after exercise, subjects received 0.5 g·kg^−1^ BW KE/PL (38 ± 1.4 mL). Thereafter, the KE/PL was delivered at a rate of 0.25 g·kg^−1^ BW·h^−1^ (19 ± 0.7 mL). This regimen was chosen to maintain high circulating plasma ketone concentrations throughout recovery (Clarke et al., 2012b).

**Table 1:** **Composition of recovery beverages**.

	**Initial drinks**	**Maintenance drinks**
**CARBOHYDRATES**		
Maltodextrin	35 (30–40)	25 (22–29)
Dextrose	19 (16–22)	13 (12–15)
Total	54 (47–62)	38 (33–44)
**Protein**		
Non-essential amino acids	7 (6–8)	6 (5–7)
Essential amino acids^*^	6 (5–7)	5.4 (5–6)
Total	14 (11–15)	12 (10–13)

*Data are means and range (in parentheses) and represent grams of ingredients for recovery drinks, which were ingested relative to body weight. A 250 ml drink was given immediately after exercise, with maintenance drinks (250 ml) given at 30 min intervals throughout the 5 h recovery period*.

* *The protein mixture administered yielded a total leucine intake of 11–14 g during the 5 h recovery period, depending on BW*.

#### Analysis of blood samples

Capillary blood samples were immediately assayed for blood glucose and β-hydroxybutyrate (Glucomen Lx plus-meter with Lx glucose or Lx β-ketone strips, Menarini Diagnostics, Firenze, Italy), and blood lactate concentrations (Lactate-Pro 2, Arkray, Kyoto, Japan). The detection limits for β-hydroxybutyrate were 0.1–8.0 millimolar (mM). Venous blood samples were immediately centrifuged to separate plasma which was stored at −80°C until assayed by ELISA for insulin concentration using a commercially available kit (catalog number: 10-1132-01, Mercodia Uppsala, Sweden).

#### Muscle biopsy procedure

Muscle biopsies were obtained immediately before and after exercise and 90 and 300 min post-exercise from the vastus lateralis of the quadriceps muscle under local anesthetic (2% xylocaine without epinephrine, 1 mL subcutaneously) using a Bergström-type needle. After removal of visible blood and non-muscle material, the muscle biopsy was frozen in isopentane cooled in liquid nitrogen. After freezing, the muscle samples were immediately stored at −80°C until later analysis.

#### Analysis of muscle samples

Muscle glycogen was measured as glucose residues after acid hydrolysis in freeze-dried muscle tissue using a standard enzymatic fluorometric assay (Lowry and Passonneau, 1972). Western blots were performed as detailed previously (Masschelein et al., 2014). In brief, frozen muscle tissue was homogenized with a Polytron mixer (Polytron Technologies, Taoyuan City, Taiwan) in ice-cold lysis buffer [1:10, w/v; 50 mM Tris-HCl, pH 7.0; 270 mM sucrose; 5 mM EGTA; 1 mM EDTA; 1 mM sodium orthovanadate; 50 mM glycerophosphate; 5 mM sodium pyrophosphate; 50 mM sodium fluoride; 1 mM dithiothreitol; 0.1% Triton X-100; and a complete protease inhibitor tablet (Roche Applied Science, Vilvoorde, Belgium)]. Homogenates were then centrifuged and supernatant was collected and stored immediately at −80°C. The protein concentration was measured using a DC protein assay kit (Bio-Rad Laboratories, Nazareth, Belgium). Proteins (15–45 μg) were separated by SDS-PAGE (8–12% gels) and transferred to polyvinylidene difluoride membranes. Subsequently, membranes were blocked in TBS-T (tris-buffered saline with Tween-20) containing 5% non-fat milk for 1 h. The following antibodies were added and incubated overnight at 4°C in TBS-T containing 5% BSA (1:1,000): phospho-Akt/PKB Ser^473^, total Akt/PKB, phospho-AMPK Thr^172^, total AMPK, phospho-S6K1 Thr^389^, total S6K1, total 4E-BP1 (Cell Signaling, Leiden, The Netherlands). Membranes were then incubated for 1 h at room temperature in secondary antibody conjugated to horseradish peroxidase (1:5,000) in TBS-T containing 5% non-fat milk. Membranes were scanned and quantified with Gene Snap and Gene Tools software (Syngene, Cambridge, UK), respectively. Results are presented as the ratio protein of interest/total form or as the ratio phosphorylated γ form/total signal. Values were expressed relative to the mean value of the post-exercise sample of their respective condition.

### *In vitro* experiments in myoblasts

C_2_C_12_ murine skeletal muscle myoblasts (ATCC, Manassas, US) were grown in DMEM (Life Technologies, Merelbeke, Belgium) supplemented with 10% fetal bovine serum, 50 U/ml penicillin and 50 μg/ml streptomycin at 37°C and 5% CO_2_. At 80% confluence, myogenic differentiation was induced by changing the growth medium to DMEM supplemented with 2% horse serum, 50 U/ml penicillin, and 50 μg/ml streptomycin. After 96 h of differentiation, plates were incubated for 30 min in DMEM containing 5.0 mM glucose and lacking leucine (Sigma-Aldrich, Bornem, Belgium). Next, cells were incubated with leucine (1.5 mM), 3-hydroxybutyrate/ hydroxybutyric acid (4 mM) and lithium-acetoacetate (1.4 mM), alone or in combination. Since KE has been shown to raise blood βHB:AcAc levels in a 4:1 ratio (Clarke et al., 2012b), the concentrations used in our *in vitro* experiments closely resemble the *in vivo* setting. Unstimulated cells served as control. After 30 min of incubation, cells were lysed and prepared for western blotting as described above. Western blot analyses were performed for, total 4E-BP1 p-S6K1^Thr389^ and total S6K1. Antibodies used were identical as for the *in vivo* experiments. In a follow-up experiment, 1 μM puromycin, a structural analog of tyrosyl-tRNA, was added to the wells during 30 min incubation at 5 mM leucine or 1.5 mM leucine, alone or together with 4 mM 3-hydroxybutyrate and 1.4 mM lithium-acetoacetate. Subsequently, cells were lysed and prepared for western blotting as described above. Western blot analyses were performed using an anti-puromycin antibody (Merck, Overijse, Belgium) diluted 1:25,000 in TBS-T containing 5% non-fat milk, to detect puromycin incorporation into nascent peptide chains (Goodman et al., 2011). Results are reported as the ratio puromycine/coomassie blue. Values were presented relative to the mean value of the unstimulated condition. All experiments were repeated at least once and each experiment consisted of three technical replicates. Leucine, lithium-acetoacetate and 3-hydroxybutyrate were purchased from Sigma-Aldrich (Bornem, Belgium).

### Statistics

Differences between mean values over time and between conditions for the study in healthy volunteers were analyzed by 2-way repeated-measures analysis of variance (GraphPad Prism, La Jolla California, USA), using time and treatment as factors. When significant, Sidak's multi comparison test was used as a *post-hoc* test. In the figures, ^*^ indicates significance for KE vs. PL within a time point, and # indicates significance vs. time point 0 within a treatment. A supplementary table (Supplementary Table 1) with the complete overview of statistical analyses has been included in this manuscript. For *in vitro* experiments, a one-way ANOVA was performed to assess the statistical significance of differences between conditions (GraphPad Prism, La Jolla California USA). When appropriate, a Tukey *t*-test was used as a *post-hoc* test. In the figures, ^*^ indicates significance vs. leucine stimulated condition, and # indicates significance vs. unstimulated condition. A supplementary table (Supplementary Table 2) with the complete overview of statistical analyses has been included in this manuscript.

A *p* < 0.05 was considered statistically significant. Data are presented as mean ± SEM.

## Results

### Study in healthy volunteers

#### Blood biochemistry

**Figure 2:**
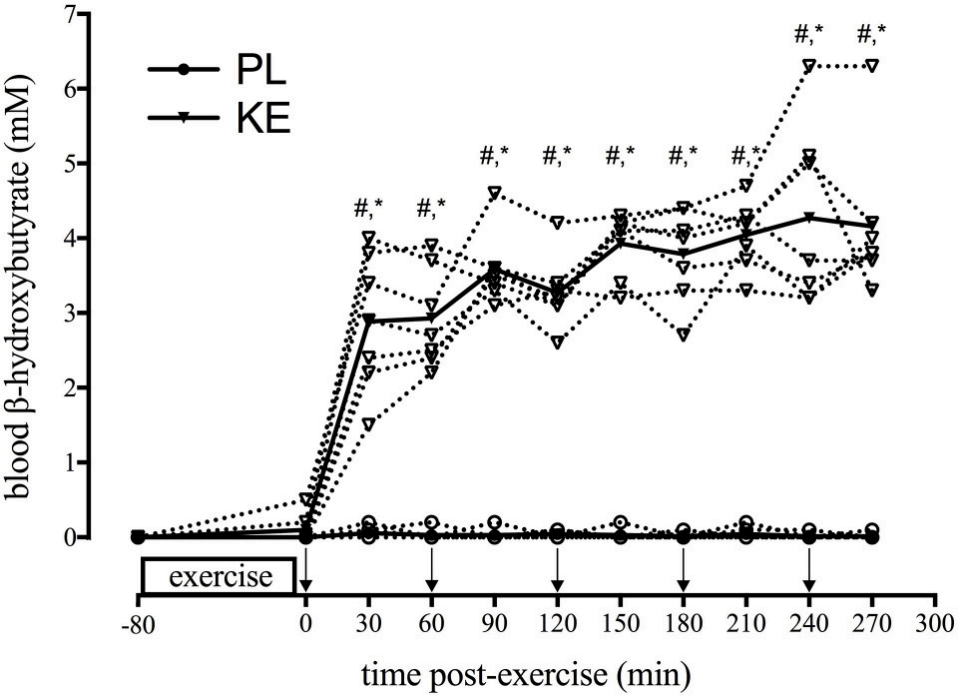
**Effect of KE intake on blood β-hydroxybutyrate**. Data points represent individual values (open symbols-dotted line) and means (filled symbols-solid line) for blood βHB concentration at baseline (−80) and during the 5 h recovery period. During recovery, either PL or KE was ingested together with a high-dose protein-carbohydrate solution. Arrows (↓) indicate ingestion of PL or KE. ^*^p < 0.05 KE vs. PL at time points indicated; ^#^*p* < 0.05 vs. 0 min post-exercise for KE.

**Effect of KE intake on blood β-hydroxybutyrate (Figure 2)**

Blood β-hydroxybutyrate (βHB) was undetectable (<0.01 mM) in PL and at baseline in KE (*p* < 0.05). Within 30 min of KE intake, blood βHB levels increased to 2.9 ± 0.3 mM with KE drinks every 60 min, gradually increased during the recovery period to reach a peak of 4.3 ± 0.5 mM at 4 h (*p* < 0.05 vs. 30 min). Blood βHB was consistently elevated from the start to the end of the recovery period, in every subject (mean:4.2 mM; range: 3.3–6.3 mM).

**Figure 3:**
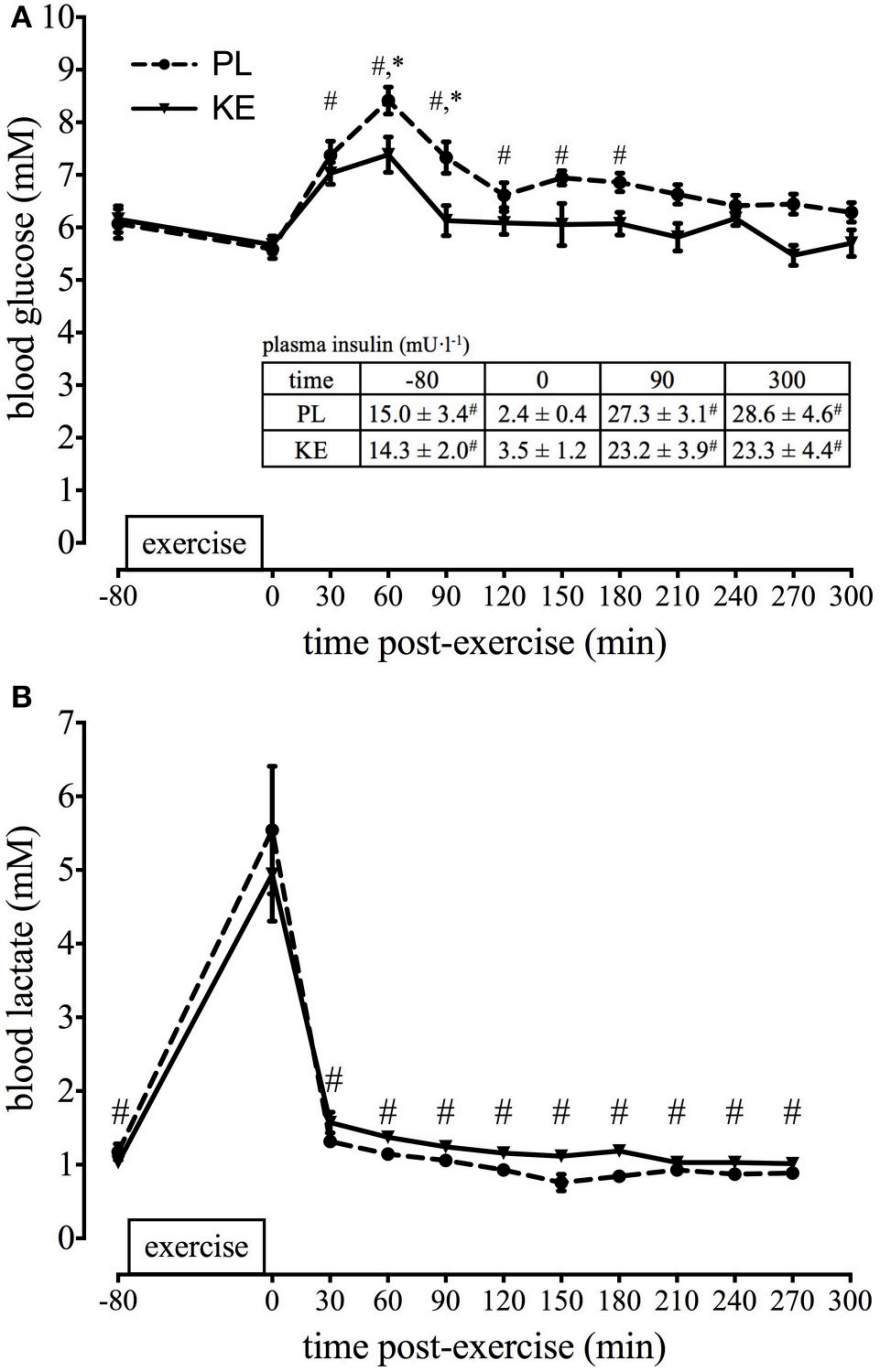
**Effect of nutritional ketosis on blood glucose, insulin, and lactate**. Data are means ± SEM (*n* = 7) and represent **(A)** blood glucose and **(B)** blood lactate at baseline (−80) and during the 5 h recovery period. Insert table shows the corresponding plasma insulin concentrations. During recovery, either PL or KE was ingested together with a high-dose protein-carbohydrate solution. ^*^p < 0.05 KE vs. PL at time points indicated; ^#^*p* < 0.05 vs. 0 min post-exercise for both KE and PL.

**Effect of nutritional ketosis on blood glucose, insulin, and lactate (Figure 3).**

Due to the high-dose carbohydrate intake blood glucose rapidly increased following exercise (*p* < 0.05), reaching peak levels in both experimental conditions at 1 h. After 1 h, despite carbohydrate drinks every 30 min, blood glucose gradually returned toward the baseline (*p* < 0.05) although blood glucose was consistently (~1 mM) lower in KE (*p* < 0.05) compared with PL. Nonetheless, plasma insulin concentrations were similar between KE and PL throughout the experiment. Exercise decreased plasma insulin from ~15 mU·l^−1^ at baseline to ~3 mU·l^−1^ (*p* < 0.05) immediately post-exercise, after which recovery insulin increased (*p* < 0.05) to ~25 mU·l^−1^ at both 90 and 300 min, irrespective of the experimental group. The knee-extension exercise increased blood lactate level to ~5 mM in both groups (*p* < 0.05). Following exercise, lactate values returned to baseline within 1 h.

#### Muscle biochemistry

**Figure 4:**
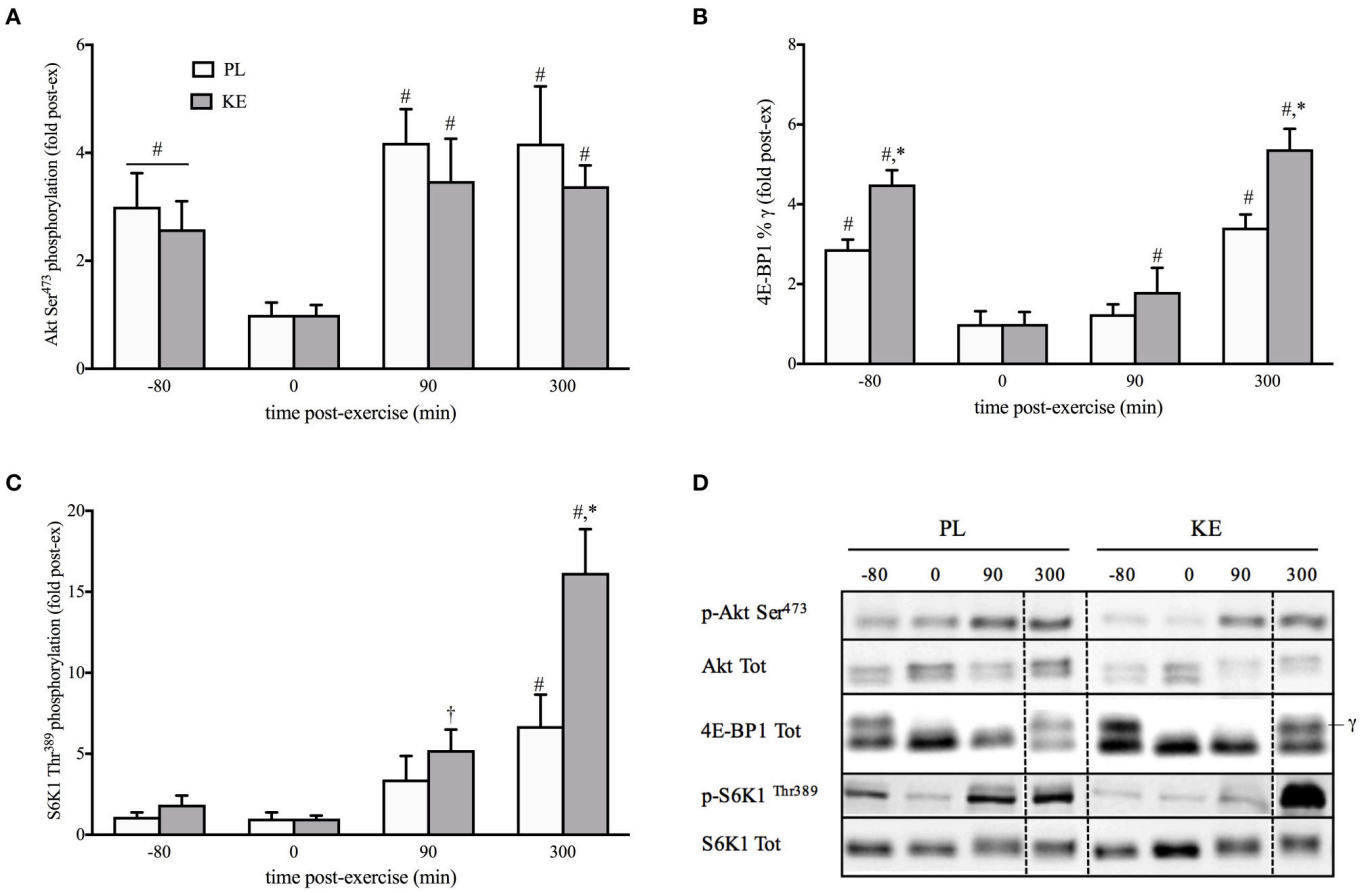
**Effect of nutritional ketosis on the Akt/mTORC1 pathway**. Data are means ± SEM (*n* = 7) for Akt **(A)**, 4E-BP1 **(B)**, and S6K1 **(C)** phosphorylation measured in m. vastus lateralis at baseline (−80) and after 0, 90, and 300 min of recovery. **(D)** Representative blots; vertical dotted line indicates a repositioned gel lane from the same blot. During recovery, either PL or KE was ingested together with a high-dose protein-carbohydrate solution ^*^p < 0.05 KE vs. PL at time points indicated; ^#^*p* < 0.05, ^†^*p* < 0.10 vs. 0 min post-exercise at conditions indicated.

**Effect of nutritional ketosis on the Akt/mTORC1 pathway (Figure 4)**

Upstream of mTORC1 and unaltered by KE, the phosphorylation status of Akt at Ser^473^ was decreased by exercise (*p* < 0.05), and returned to baseline within 90 min of recovery. To assess mTORC1 activity, we assessed the phosphorylation status of P70^S6k1^ at Thr^389^ (p-S6K1^Thr389^), and the percentage of 4E-BP1 in the phosphorylated γ-form (4E-BP1%γ), two of its main downstream targets involved in protein synthesis. Exercise decreased 4E-BP1%γ (*p* < 0.05), but not p-S6K1^Thr389^, for both KE and PL. During recovery, p-S6K1^*Thr*389^ and 4E-BP1%γ gradually increased (*p* < 0.05), with the increase greater in KE than in PL. Thus, compared with PL at the end of the 5 h recovery period, p-S6K1^Thr389^ (~2.5-fold) and 4E-BP1%γ (~60%) were higher in KE (*p* < 0.05). Total protein contents of Akt, S6K1, and 4E-BP1 were not different between groups, nor exhibited a change across time.

**Figure 5:**
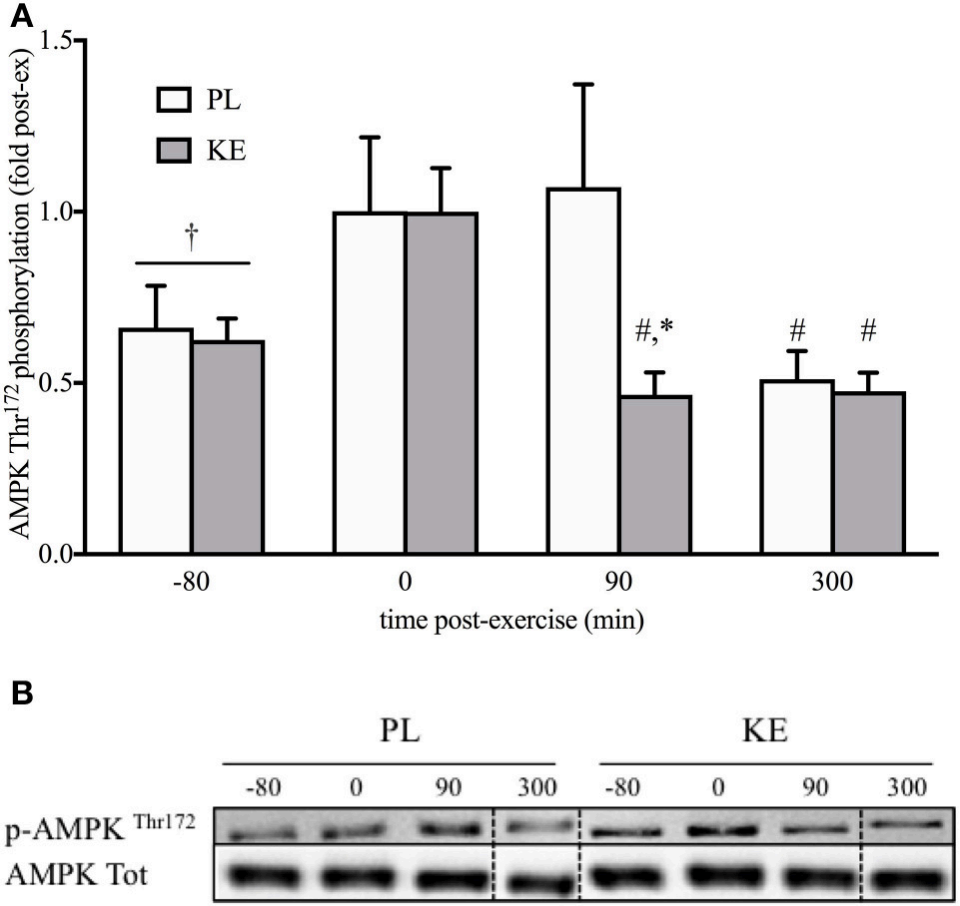
**Effect of nutritional ketosis on AMPK^**Thr172**^ phosphorylation**. Data are means ± SEM (*n* = 7) and represent **(A)** AMPK phosphorylation measured in m. vastus lateralis at baseline (−80) and after 0, 90, and 300 min of recovery. **(B)** Representative blots; vertical dotted line indicates a repositioned gel lane from the same blot. During recovery, either PL or KE was ingested together with a high-dose protein-carbohydrate solution. ^*^p < 0.05 KE vs. PL at time points indicated; ^#^*p* < 0.05, ^†^*p* < 0.10 vs. 0 min post-exercise at conditions indicated.

**Effect of nutritional ketosis on AMPKThr172 phosphorylation (Figure 5)**

Exercise did not significantly increase (*p* = 0.08) AMPK phosphorylation at Thr^172^ (p-AMPK^Thr172^). p-AMPK^Thr172^ in KE, but not in PL, was the same as baseline within 90 min of recovery, with lower p-AMPK^Thr172^ in KE than in PL (*p* < 0.05). At the end of the recovery period, p-AMPK^Thr172^ was baseline for both KE and PL. Total protein content of AMPK was not different between groups, nor exhibited a change across time.

**Figure 6:**
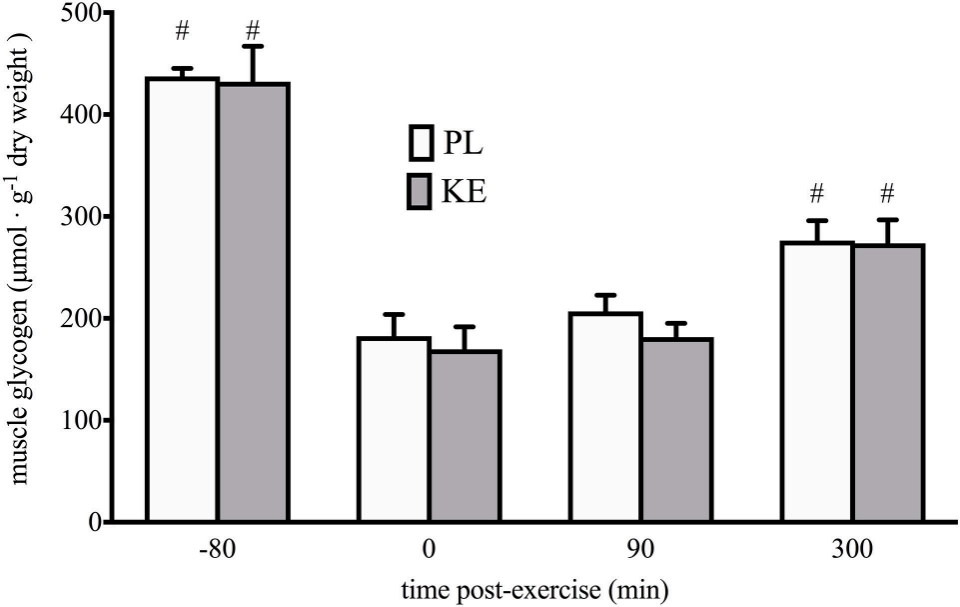
**Effect of nutritional ketosis on muscle glycogen**. Data are means ± SEM (*n* = 7) and represent muscle glycogen content measured in m. vastus lateralis at baseline (−80) and after 0, 90, and 300 min of recovery. During recovery, either PL or KE was ingested together with a high-dose protein-carbohydrate solution. ^#^*p* < 0.05 vs. 0 min post-exercise at conditions indicated.

**Effect of nutritional ketosis on muscle glycogen (Figure 6)**

Initial muscle glycogen content was 432 ± 35 μmol g^−1^ dry weight in PL, vs. 437 ± 8 μmol g^−1^ dry weight in KE. Exercise depleted muscle glycogen by ~60% (*p* < 0.05) to ~170 ± 22 and 183 ± 21 μmol. g^−1^ dry weight in PL and KE, respectively. Post-exercise glycogen resynthesis during the 5 h recovery period returned muscle glycogen contents to ~274 ± 23 in KE vs. 276 ± 20 μmol. g-1 dry weight in PL. There were no differences between PL and KE at any time.

#### Gastrointestinal tolerance

Compared with PL (6 ± 2 out of a maximum of 32), adverse upper abdominal discomfort scored higher in KE (17 ± 2, *p* < 0.05). Lower abdominal and systemic discomfort was similar between the groups. Due to upper abdominal discomfort in KE, four subjects did not ingest supplements beyond 4 h of recovery. Nonetheless, plasma βHB, blood glucose and plasma insulin levels were unaffected, i.e., values were identical between subgroups exhibiting upper abdominal discomfort vs. no symptoms.

#### *In vitro* experiments in myoblasts

**Figure 7:**
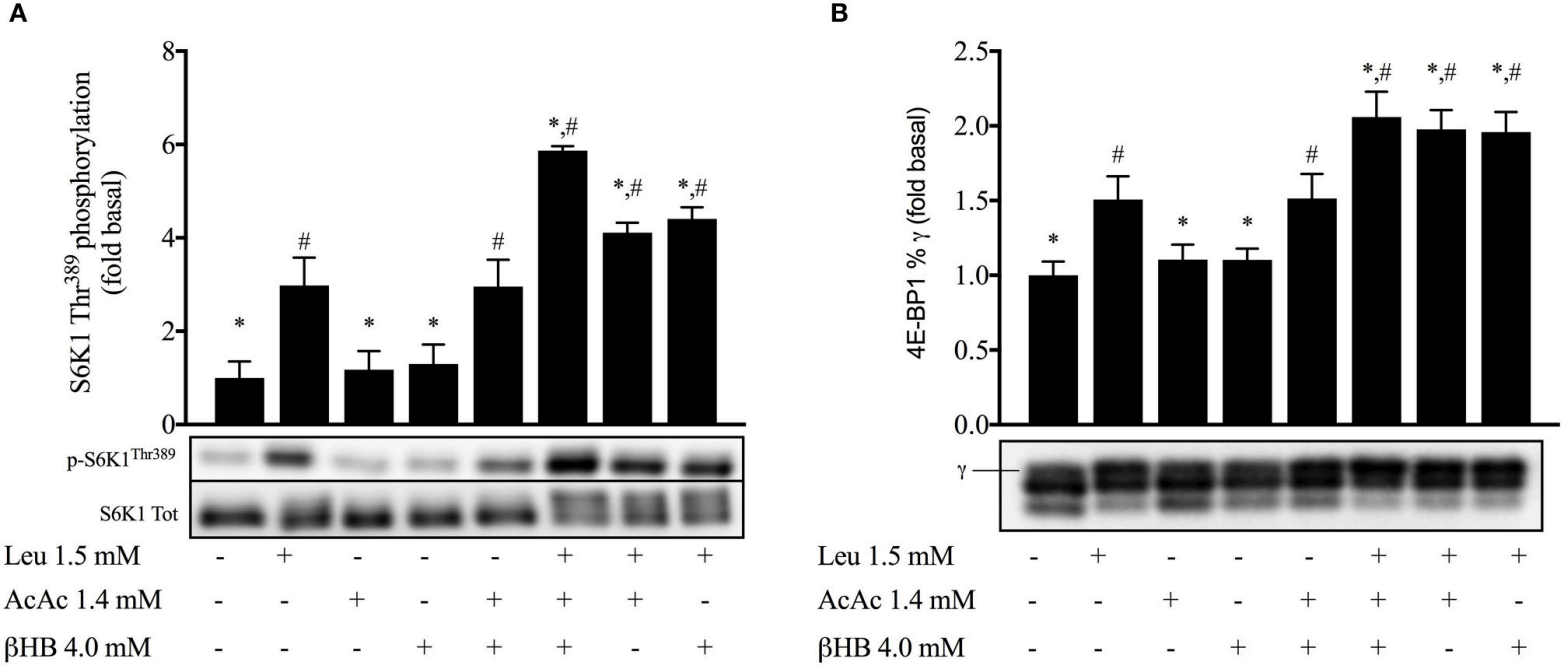
**Effect of ketones and leucine on 4E-BP1 and S6K1^**Thr389**^ phosphorylation in myotubes**. Data are means ± SEM (*n* = 3). Leu, leucine; AcAc, lithium-acetoacetate; βHB, β-hydroxybutyrate. ^*^p < 0.05 indicates significance vs. leucine stimulated condition; ^#^*p* < 0.05 indicates significance vs. unstimulated condition.

**Effect of ketones and leucine on 4E-BP1 and S6K1^Thr389^ phosphorylation in myotubes (Figure 7)**

Leucine alone increased p-S6K1^Thr389^ ~3-fold and 4E-BP1%γ by 51% (p < 0.05). Incubation with either acetoacetate or βHB alone did not increase p-S6K1^Thr389^ or 4E-BP1%γ, but the combination of both ketone bodies increased p-S6K1^Thr389^ ~ 3-fold and 4E-BP1%γ by 51% (*p* < 0.05). The addition of either acetoacetate or βHB with leucine elevated p-S6K1^Thr389^ 4-fold and 4E-BP1%γ 2-fold (*p* < 0.05). Leucine + acetoacetate + βHB increased p-S6K1^Thr389^ ~ 6-fold and 4E-BP1%γ 2-fold vs baseline, more than any of the substrates alone (*p* < 0.05).

**Figure 8:**
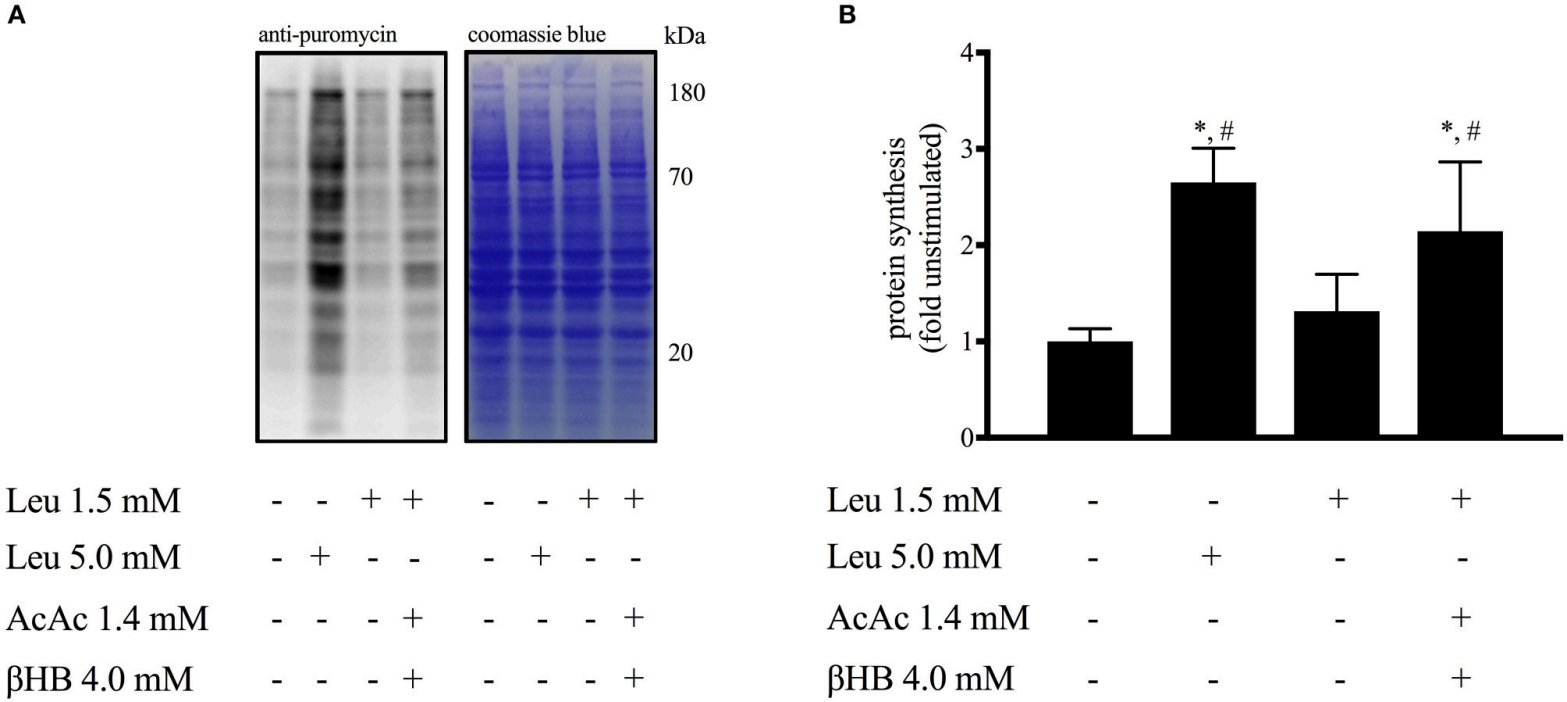
**Effect of ketones and leucine on muscle protein synthesis in myotubes**. Data are means ± SEM (*n* = 3). **(A)** Representative image of western blot analysis for puromycin (left) followed by coomassie blue staining (right) to verify equal loading of proteins. **(B)** Quantification of the puromycin-labeled peptides corrected for coomassie blue staining. Leu, leucine; AcAc, lithium-acetoacetate; βHB, β-hydroxybutyrate. ^*^p < 0.05 indicates significance vs. leucine stimulated condition; ^#^*p* < 0.05 indicates significance vs. unstimulated condition.

**Effect of ketones and leucine on protein synthesis in myotubes (Figure 8)**

Leucine (1.5 mM) alone did not alter protein synthesis rate as measured by puromycin incorporation into nascent peptide chains (Goodman et al., 2011). However, the addition of AcAc + βHB enhanced protein synthesis ~2-fold (*p* < 0.05). A supraphysiological leucine concentration (5.0 mM) was found to stimulate protein synthesis 2.5-fold (*p* < 0.05).

## Discussion

In this study, we investigated the potential of nutritional ketosis to stimulate markers of post-exercise muscle protein and glycogen synthesis, since both may enhance long-term training adaptations and facilitate muscle recovery from intense exercise (Hawley et al., 2011). We found that oral KE ingestion increased mTORC1 activation in human skeletal muscle following strenuous exercise, but left muscle glycogen resynthesis unaffected. To explore whether the increase in mTORC1 activation leads to increased protein synthesis, we subsequently performed a set of *in vitro* experiments using C_2_C_12_ myotubes. We confirmed that ketone bodies stimulated mTORC1 activity and showed that this leads to enhanced leucine-mediated muscle protein synthesis. Taken together, our results indicate that KE enhances the anabolic response to both exercise and protein ingestion but does not affect glycogen resynthesis.

We aimed to elevate plasma ketone concentrations using intermittent (60 min) drinks of a novel oral ketone ester (Clarke et al., 2012b) during the initial 5 h of recovery following a glycogen-depleting maximal exercise bout. In conjunction with KE, we also administered a mixture containing whey protein and carbohydrates in a quantity that is considered to elicit maximal rates of post-exercise muscle protein (Morton et al., 2015) and glycogen synthesis (Burke et al., 2016), because we wanted to explore whether KE could add to the recommended nutritional procedures for stimulation of muscle recovery. KE increased blood βHB concentration to ~3–5 mM within 30 min after the first drink to remain elevated to the end of the recovery period in all subjects. As KE raises blood βHB:AcAc levels in a 4:1 ratio (Clarke et al., 2012b) total plasma KB concentrations above 6 mM are conceivable, levels similar to those reported after several weeks of starvation (Cahill, 2006).

We did not detect any rise in post-exercise ketone levels in the PL condition. Post-exercise ketosis is inversely correlated to liver glycogen content (Adams and Koeslag, 1988), which in short one-leg postprandial exercise protocols is not depleted (Gonzalez et al., 2016). In addition, post-exercise nutritional intake, causing hyperinsulinemia, also reduces endogenous ketosis (Koeslag, 1982; Impey et al., 2016). Therefore, in PL post-exercise ketosis is unlikely to have significantly contributed to muscle recovery.

Muscle protein synthesis following exercise is mainly controlled by mTORC1 (Bodine et al., 2001; Schiaffino and Mammucari, 2011). Assessment of the phosphorylation status of the mTORC1 substrates S6K1 and 4EBP1 is a reliable readout of mTORC1 activity and is closely linked to muscle hypertrophy both in humans (Terzis et al., 2008) and mice (Baar and Esser, 1999). Moreover, S6K1 activation is required to increase muscle force (Marabita et al., 2016). We found the ingestion of KE to increase mTORC1 activity during recovery from exercise, as indicated by higher phosphorylation status of S6K1 and 4EBP1 (Figure 4). While evaluation of muscle protein synthesis via key proteins involved in protein translation has been shown to be a reliable procedure (Baar and Esser, 1999; Bodine et al., 2001; Terzis et al., 2008; Drummond et al., 2009), a dissociation between S6K1/4EBP-1 phosphorylation and muscle protein synthesis might still be possible (Greenhaff et al., 2008). Since our study protocol did not allow us to assess muscle protein synthesis rates in human subjects *in vivo*, we decided to set up a series of *in vitro* experiments using C_2_C_12_ myotubes. First, we confirmed that KB potentiate mTORC1 signaling following leucine stimulation (Figure 7). Subsequently, we found that the increase in mTORC1 activity was reflected by increased protein synthesis in muscle cells *in vitro* (Figure 8). While our *in vitro* data show the ability of KB to potentiate leucine-mediated protein synthesis in myotubes, further human studies using stable isotope tracers need to confirm whether or not KE can also promote muscle protein synthesis in humans.

The ability for KE to promote mTORC1 activity occurred in the absence of increased blood insulin levels and downstream insulin signaling pathway. Moreover, we could reproduce these effects using strictly controlled *in vitro* settings. This indicates that the effect of KE/KB is independent of growth factor signaling as well as potential other systemic effects induced by ketosis (Egan and D'Agostino, 2016; Holdsworth et al., 2016).

While we for the first time show that KE stimulates mTORC1 signaling post-exercise against the background of high-rate protein plus carbohydrate intake in humans, it is well established that KB modulate muscle protein metabolism during starvation. When glucose availability is low, KB replace glucose as the primary brain fuel, and therefore suppresses the need for muscle proteolysis to provide gluconeogenic precursors (Sherwin et al., 1975; Owen, 2005). Furthermore, hyperketonaemia induced by intravenous infusions of a ketone salt after an overnight fast, decreased leucine oxidation and increased protein synthesis in humans (Nair et al., 1988). The direct effect of KB on protein synthesis rates in muscle has only been explored in a limited number of *ex vivo* studies. One study found physiological concentrations of KB without amino acids in the incubation medium not to stimulate protein synthesis in myocytes (Tischler et al., 1982).

Another *ex vivo* study tested the effect of either 4 mM βHB or 4 mM AcAc in chicken muscles and reported a decrease in protein synthesis. However, raising glutamine availability restored protein synthesis (Wu and Thompson, 1990). By analogy, our results show a synergistic effect of physiological concentrations of ketone bodies and leucine on S6K1 phosphorylation (Figure 7) and a direct effect of ketone bodies on leucine-mediated protein synthesis (Figure 8). Taken together these results indicate the potential of ketone bodies to stimulate muscle protein anabolism.

Although we are unable to identify the precise mechanism responsible for enhanced muscle protein synthesis with KE, it is known that skeletal muscle protein synthesis is reduced when energy homeostasis is impaired (Hardie, 2011). Following high-intensity exercise, activation of AMPK is responsible for the delayed activation of muscle protein synthesis (Dreyer et al., 2006; Thomson et al., 2008). Rapid restoration of muscle energy balance to de-activate AMPK is therefore important to initiate post-exercise mTORC1 activation and protein synthesis. KE ingestion markedly promoted the decrease in AMPK activation early during recovery, potentially via serving as an additional energy substrate for oxidative ATP production (Cox et al., 2016; Murray et al., 2016) which might allow faster recovery of energy homeostasis and facilitate mTORC1 activation.

Nutritional ketosis was recently shown to alter substrate competition during endurance exercise and to reduce glycolysis, thereby preserving glycogen availability (Cox et al., 2016). Early experiments in dogs and rats have shown that ketosis can stimulate muscle glucose uptake (Madison et al., 1964) as well as glycogen storage (Maizels et al., 1977). Furthermore, ketosis generated by either a chronic high-fat, low-carbohydrate diet (Phinney et al., 1983) or by KB intake (Cox et al., 2016) decreased glycogen breakdown both at rest (Cox et al., 2016) and during exercise (Phinney et al., 1983; Cox et al., 2016). On the other hand, inducing ketosis by adhering to a ketogenic diet did not affect glycogen repletion patterns after a 3-h run (Volek et al., 2016). Based on these data, we hypothesized that during recovery from high-intensity exercise, KE ingestion could promote glycogen resynthesis by reducing disposal of glucose units via glycolytic breakdown. Suppression of glycolysis increases glucose-6-phosphate availability, which is a potent stimulator of the glycogen synthase enzyme (Jensen and Richter, 2012). However, contrary to our hypothesis, we found KE not to affect glycogen resynthesis during the 5 h recovery period. These data are in apparent contradiction with the results of another study in which KE increased muscle glycogen resynthesis following glycogen-depleting exercise (Holdsworth et al., 2016). In this study, however, CHO was delivered intravenously during a 2 h hyperglycaemic clamp. Under those conditions, KE ingestion in combination with carbohydrates resulted in 2-fold higher plasma insulin levels compared to carbohydrates alone. This was conceivably due to stimulation of pancreatic insulin secretion by high circulating plasma KB (Balasse et al., 1970; Beylot et al., 1986). Conversely, high-rate carbohydrate intake in conjunction with proteins in the conditions of the current study resulted in substantially higher plasma insulin levels than in the aforementioned studies due to the synergistic action of glucose and amino acids on pancreatic insulin release (Floyd et al., 1970). In addition, plasma insulin was identical between KE and PL (Figure 3A).

In the current study, subjects ingested 1.5 g·kg^−1^ BW KE, concentrations that have been reported previously to efficiently enhance blood ketone levels over longer time periods (Clarke et al., 2012b; Cox et al., 2016). In agreement with these studies, intermittent ingestion of KE with a protein-carbohydrate mixture resulted in blood βHB levels of ~5 mM but also resulted in gastrointestinal discomfort in half of the subjects. Gastrointestinal issues have been reported before under nutritional conditions not involving concomitant high-rate protein and carbohydrate intake (Clarke et al., 2012b). Although we cannot exclude that GI discomfort might have affected our experiment, biochemical measurements and circulating concentrations of βHB, glucose, and insulin were identical between subjects experiencing GI distress and subjects with adequate GI comfort. Future studies will need to elucidate more optimal dosing strategies to maintain the effect of KE on muscle protein synthesis in the absence of GI complaints.

In conclusion, while ingestion of KE did not increase muscle glycogen resynthesis, it markedly increased mTORC1 signaling following high-intensity exercise. Using C_2_C_12_ myotubes, we confirmed that KB potentiated leucine mediated mTORC1 activation and that this resulted into higher leucine-mediated protein ynthesis. Our results indicate that KE enhances the anabolic response to both exercise and protein ingestion but does not affect muscle glycogen synthesis, suggesting that it might become an attractive nutritional strategy to increase training-induced muscle remodeling and adaptation.

## Ethics statement

The study was approved by the KU Leuven Biomedical Ethics Committee (B322201316517) and was conducted in accordance with the Declaration of Helsinki. All participants provided written informed consent after clearing medical screening and being fully informed about the content of the experiments and the risks involved.

## Author contributions

Conception and design of the study: PH, TV, and SD. All authors contributed to the acquisition, analysis and interpretation of the data, and revised and approved the final manuscript for important intellectual content written by TV, PH, and KD.

## Funding

This work was supported by the Flemish Ministry of Sport, BLOSO—“Leerstoel Topsport Inspanningsfysiologie.” Research Fund Flanders (FWO) supports TV by an FWO-fellowship strategic basic research (1S60116N).

### Conflict of interest statement

The intellectual property and patents covering the use of the (R)-3-hydroxybutyl (R)-3-hydroxybutyrate ketone mono-ester are owned by BTG Ltd., The University of Oxford, the NIH and TdeltaS Ltd. KC is a non-executive director of TdeltaS, a spin out company of the University of Oxford. All other authors declare that the research was conducted in the absence of any commercial or financial relationships that could be construed as a potential conflict of interest.

## Abbreviations

AcAc: aceto-acetate
AMPK: AMP-activated protein kinase
BSA: bovine serum albumin
βHB: beta-hydroxybutyrate
BW: body weight
DMEM: Dulbecco's Modified Eagle Medium
4E-BP1: eukaryotic initiation factor 4E-binding protein 1
KB: ketone bodies
KE: ketone ester [(R)-3-hydroxybutyl (R)-3-hydroxybutyrate]
mM: millimolar
mTORC1: mechanistic target of rapamycin complex 1
PL: placebo
1RM: one-repetition max
S6K1: p70 ribosomal S6 kinase 1
TBS-T: tris-buffered saline with Tween-20
TCA: tricarboxylic acid cycle
TSC2: tuberous sclerosis complex 2
kDa: kilodalton.
