# Supplementary material for: Intake of a Ketone Ester Drink during Recovery from Exercise Promotes mTORC1 Signaling but Not Glycogen Resynthesis in Human Muscle
Source: Front Physiol. 2017 May 23;8:310. doi: 10.3389/fphys.2017.00310 (PMC5440563; doi:10.3389/fphys.2017.00310)
Supplement: Supplementary file 1 [file Table1.DOCX]

|  | *2-way ANOVA*  *(time x treatment)* | *Sidak’s multiple comparisons test* | | | |
| --- | --- | --- | --- | --- | --- |
|  |  | *Time* | | | *Treatment* |
|  |  | *PL* | *KE* | *PL+KE* |  |
| *p-Akt^Ser473^* | Interaction: 0.9168  Time: <0.0001  Treatment: 0.2738 | 0 Vs. -80: 0.0649  0 Vs. 90: 0.0015  0 Vs. 300: 0.0017 | 0 Vs. -80: 0.1791  0 Vs. 90: 0.0165  0 Vs. 300: 0.0223 | 0 Vs. -80: 0.0153  0 Vs. 90: <0.0001  0 Vs. 300: <0.0001 | -80 (PL Vs. KE): 0.9814  0 (PL Vs. KE): >0.9999  90 (PL Vs. KE): 0.8846  300 (PL Vs. KE): 0.8395 |
| *4E-BP1%γ* | Interaction: 0.0002  Time: <0.0001  Treatment: 0.0472 | 0 Vs. -80: <0.0001  0 Vs. 90: 0.8184  0 Vs. 300: <0.0001 | 0 Vs. -80: <0.0001  0 Vs. 90: 0.0425  0 Vs. 300: <0.0001 | 0 Vs. -80: <0.0001  0 Vs. 90: 0.0669  0 Vs. 300: <0.0001 | -80 (PL Vs. KE): 0.0170  0 (PL Vs. KE): >0.9999  90 (PL Vs. KE): 0.7713  300 (PL Vs. KE): 0.0027 |
| *p-S6K1^Thr389^* | Interaction: 0.0033  Time: <0.0001  Treatment: 0.0182 | 0 Vs. -80: 0.9999  0 Vs. 90: 0.4963  0 Vs. 300: 0.0124 | 0 Vs. -80: 0.9561  0 Vs. 90: 0.0860  0 Vs. 300: <0.0001 | 0 Vs. -80: 0.9764  0 Vs. 90: 0.0485  0 Vs. 300: <0.0001 | -80 (PL Vs. KE): 0.9923  0 (PL Vs. KE): >0.9999  90 (PL Vs. KE): 0.8288  300 (PL Vs. KE): <0.0001 |
| *p-AMPK^Thr172^* | Interaction: 0.1029  Time: 0.0064  Treatment: 0.2261 | 0 Vs. -80: 0.2480  0 Vs. 90: 0.9786  0 Vs. 300: 0.0498 | 0 Vs. -80: 0.1789  0 Vs. 90: 0.0288  0 Vs. 300: 0.0327 | 0 Vs. -80: 0.0415  0 Vs. 90: 0.2755  0 Vs. 300: 0.0024 | -80 (PL Vs. KE): 0.9997  0 (PL Vs. KE): >0.9999  90 (PL Vs. KE): 0.0281  300 (PL Vs. KE): 0.9997 |
| *Muscle glycogen* | Interaction: 0.8560  Time: <0.0001  Treatment: 0.6465 | 0 Vs. -80: <0.0001  0 Vs. 90: 0.5493  0 Vs. 300: 0.0002 | 0 Vs. -80: <0.0001  0 Vs. 90: 0.9102  0 Vs. 300: <0.0001 | 0 Vs. -80: <0.0001  0 Vs. 90: 0.5041  0 Vs. 300: <0.0001 | -80 (PL Vs. KE): 0.9997  0 (PL Vs. KE): 0.9877  90 (PL Vs. KE): 0.8734  300 (PL Vs. KE): >0.9999 |

*Supplementary Table 1. Additional statistical information In vivo experiments.*

Data are p-values for two- way repeated-measures analysis of variance (ANOVA) and Sidak’s multiple comparisons test as calculated using GraphPad Prism 7.0, La Jolla California, USA. Abbreviations: PL, placebo; KE, ketone ester; KE, ketone ester (R)-3-hydroxybutyl (R)-3-hydroxybutyrate; ANOVA, analysis of variance.
