# Supplementary material for: Intake of a Ketone Ester Drink during Recovery from Exercise Promotes mTORC1 Signaling but Not Glycogen Resynthesis in Human Muscle
Source: Front Physiol. 2017 May 23;8:310. doi: 10.3389/fphys.2017.00310 (PMC5440563; doi:10.3389/fphys.2017.00310)
Supplement: Supplementary file 2 [file Table2.DOCX]

|  | *1-way ANOVA* | | *Tukey's multiple comparisons test* | | |
| --- | --- | --- | --- | --- | --- |
|  | *p-S6K1^Thr389^* | *4E-BP1%γ* |  | *p-S6K1^Thr389^* | *4E-BP1%γ* |
| *mTORC1 activation* | P<0.0001 | P<0.0001 | Unstimulated Vs. Leu | 0.0003 | 0.0045 |
|  |  |  | Unstimulated Vs. AcAc | 0.9989 | 0.9711 |
|  |  |  | Unstimulated Vs. βHB | 0.9765 | 0.9733 |
|  |  |  | Unstimulated Vs. AcAc+βHB | 0.0010 | 0.0040 |
|  |  |  | Unstimulated Vs. AcAc+βHB + Leu | <0.0001 | <0.0001 |
|  |  |  | Unstimulated Vs. AcAc+ Leu | <0.0001 | <0.0001 |
|  |  |  | Unstimulated Vs. βHB + Leu | <0.0001 | <0.0001 |
|  |  |  | Leu Vs. AcAc | 0.0007 | 0.0301 |
|  |  |  | Leu Vs. βHB | 0.0014 | 0.0293 |
|  |  |  | Leu Vs. AcAc+βHB | >0.9999 | >0.9999 |
|  |  |  | Leu Vs. AcAc+βHB+Leu | <0.0001 | 0.0020 |
|  |  |  | Leu Vs. AcAc +Leu | 0.0379 | 0.0087 |
|  |  |  | Leu Vs. βHB +Leu | 0.0066 | 0.0123 |
|  |  |  | AcAc Vs. βHB | >0.9999 | >0.9999 |
|  |  |  | AcAc Vs. AcAc+βHB | 0.0024 | 0.0268 |
|  |  |  | AcAc Vs. AcAc+βHB+Leu | <0.0001 | <0.0001 |
|  |  |  | AcAc Vs. AcAc+Leu | <0.0001 | <0.0001 |
|  |  |  | AcAc Vs. βHB +Leu | <0.0001 | <0.0001 |
|  |  |  | βHB Vs. AcAc+βHB | 0.0045 | 0.0261 |
|  |  |  | βHB Vs. AcAc+βHB+Leu | <0.0001 | <0.0001 |
|  |  |  | βHB Vs. AcAc+Leu | <0.0001 | <0.0001 |
|  |  |  | βHB Vs. βHB +Leu | <0.0001 | <0.0001 |
|  |  |  | AcAc+βHB Vs. AcAc+ βHB+Leu | <0.0001 | 0.0022 |
|  |  |  | AcAc+βHB Vs. AcAc +Leu | 0.0682 | 0.0097 |
|  |  |  | AcAc+βHB Vs. βHB +Leu | 0.0145 | 0.0139 |
|  |  |  | AcAc+βHB+Leu Vs. AcAc +Leu | 0.0009 | 0.9929 |
|  |  |  | AcAc+βHB+Leu Vs. βHB +Leu | 0.0053 | 0.9762 |
|  |  |  | AcAc+ Leu Vs. βHB +Leu | 0.9770 | >0.9999 |

|  | *1-way ANOVA* | *Tukey's multiple comparisons test* | |
| --- | --- | --- | --- |
| *Proteine synthese* | P<0.0001 | Unstimulated Vs. Leu 5 mM | <0.0001 |
|  |  | Unstimulated Vs. Leu 1.5 mM | 0.6794 |
|  |  | Unstimulated Vs. AcAc+βHB + Leu 1.5 mM | 0.0029 |
|  |  | Leu 5 mM Vs. Leu 1.5 mM | 0.0006 |
|  |  | Leu 5 mM Vs. AcAc + βHB + Leu | 0.2940 |
|  |  | Leu 1.5 mM Vs AcAc + βHB + Leu | 0.0350 |

*Supplementary Table 2. Additional statistical information In vitro experiments.*

Data are p-values for one- way analysis of variance (ANOVA) and Tukey’s multiple comparisons test as calculated using GraphPad Prism 7.0, La Jolla California, USA. Abbreviations: Leu; leucine, AcAc; lithium-acetoacetate, βHB; β-hydroxybutyrate.
